# CT Imaging Findings of Porcelain Gallbladder and Epiploic Appendagitis: Two Rare Abdominal Pathologies in an Asymptomatic Patient

**DOI:** 10.7759/cureus.15578

**Published:** 2021-06-10

**Authors:** Jarod Shelton, Shawn Terry

**Affiliations:** 1 General Surgery, Wellspan York Hospital, York, USA; 2 Trauma & Critical Care Surgery, Wellspan York Hospital, York, USA

**Keywords:** porcelain gallbladder, epiploic appendagitis, general surgery, resident, surgical residency, acute abdomen, abdominal pain, incidental radiological finding, gallbladder carcinoma

## Abstract

Porcelain gallbladder (PG) and epiploic appendagitis (EA) are rare imaging findings in an asymptomatic patient. The clinical presentation of PG and EA can vary; however, a common presenting complaint is abdominal discomfort. We describe the case of a 54-year-old male with computerized tomography findings consistent with PG and EA. We also performed a review of the literature to understand the etiology, clinical presentation, and diagnosis and treatment options of both PG and EA.

## Introduction

Porcelain gallbladder (PG) refers to calcification of the inner gallbladder wall while epiploic appendagitis (EA) refers to either spontaneous torsion of an epiploic appendage or venous thrombosis of a draining appendageal vein. PG and EA are rare imaging findings, especially in a patient with a benign abdomen. Although the true incidence of (EA) is likely underestimated, it has been reported at 8.8 per 1 million people[1]. EA typically presents with focal abdominal pain that is self-limited and spontaneously resolves within two weeks [2]. Classically, patients with EA may have been subjected to surgery to identify the etiology of their abdominal pain and in fact, studies suggest that EA is only diagnosed correctly pre-operatively 2.5% of the time [3]. Most cases of PG, on the other hand, are asymptomatic. However, gallbladder wall thickening with either mucosal or intramural calcification are chronic events that usually present with associated gallstones and biliary colic [4]. Similar to EA, the true incidence of PG is likely underreported with current estimates being 0.06-0.08% of routine cholecystectomy specimens [5]. Prior investigations found a strong association between PG and gallbladder carcinoma, yet, new evidence suggests that the relationship is markedly lower than previously calculated (7-61.5% versus 2-3%), and not all presentations require cholecystectomy [6]. The purpose of this case report is to discuss the clinical presentation and treatment recommendations of a single patient with incidental findings of both PG and EA. This report was deemed exempt by our institutional review board. Additionally, the patient provided informed consent for their medical course to be used for educational purposes.

## Case presentation

A 54-year-old male with known hypertension, diet-controlled diabetes mellitus, cholelithiasis, and esophageal varices secondary to alcoholic cirrhosis (model for end-stage liver disease with sodium; MELD-NA of 15) presented to an outside hospital (OSH) after six episodes of hematemesis and a single episode of black, tarry stool of less than one-day duration. The patient was hospitalized five months prior for hematemesis and underwent banding of six esophageal varices. Unfortunately, the patient was not compliant with medical recommendations and continued to imbibe, resulting in worsening alcoholic cirrhosis. On presentation to the OSH, the patient’s blood pressure was 85/54 mmHg with a heart rate of 84. An electrocardiogram showed normal sinus rhythm. Home medications included 20 milligrams of propanolol two times daily with suppression of reflex tachycardia. He was initially resuscitated with 2 liters of 0.9% normal saline with an improvement of blood pressure to 114/64. His hematemesis continued during fluid resuscitation, and he was given 2 units of packed red blood cells with pre-transfusion hemoglobin of 7.8. Intravenous (IV) pantoprazole (Protonix, Pfizer), tranexamic acid, and a continuous octreotide drip were initiated. The patient’s hematemesis persisted, and he was transferred to our tertiary medical facility.

**Figure 1:**
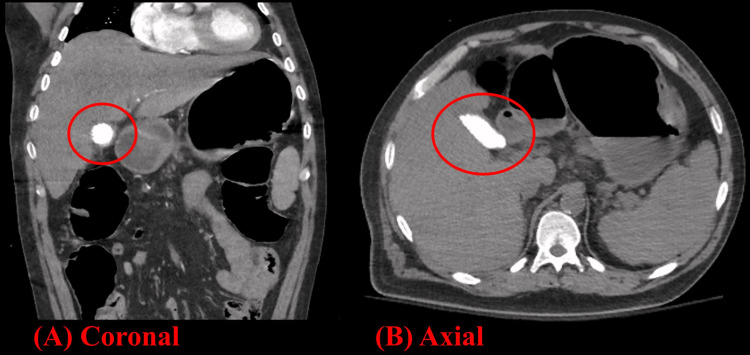
CT abdomen and pelvis coronal (A) and axial (B) images showing a diffusely radiopaque gallbladder (circle) consistent with porcelain gallbladder.

**Figure 2:**
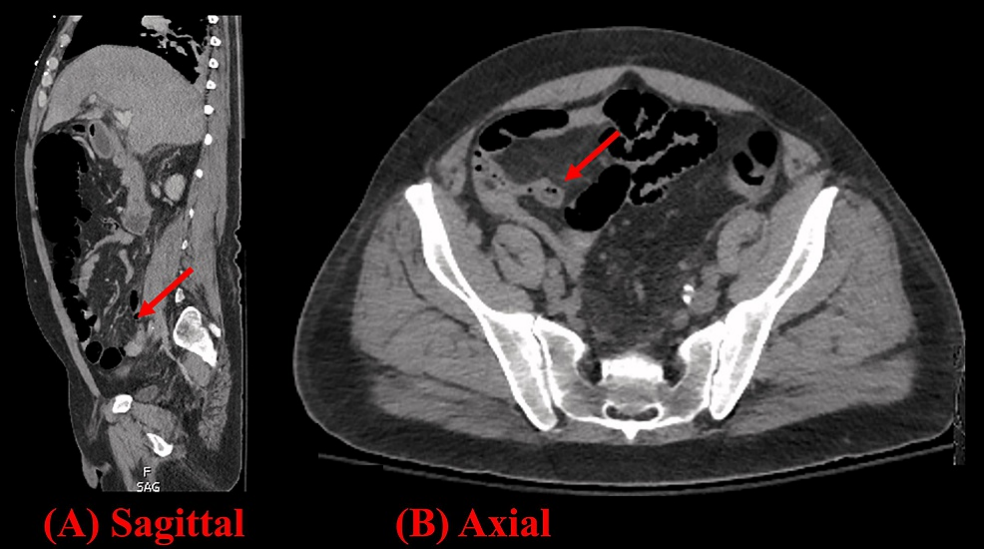
CT abdomen and pelvis coronal (A) and axial (B) images showing a fat-density, ovoid structure (arrow) adjacent to the colon with a thin high-density rim (hyperattenuating ring sign) consistent with epiploic appendagitis.

The patient was admitted to the medical intensive care unit, and gastroenterology (GI) was consulted, who recommended urgent, bedside esophagogastroduodenoscopy (EGD) to evaluate the etiology of hematemesis. He was intubated for airway protection with the chest radiograph showing good endotracheal tube position and mild pulmonary edema. He underwent EGD, which showed three chains of large esophageal varices, including a nipple sign consistent with stigmata of recent hemorrhage. The three esophageal varices were banded, which resolved the hematemesis. On post-procedure day two, the patient again experienced hematemesis while remaining hemodynamically stable, and interventional radiology (IR) was consulted for transjugular intrahepatic portosystemic shunt (TIPS). Computerized tomography (CT) of the abdomen and pelvis was ordered for pre-procedural planning with incidental findings of PG (Figure 1) and EA (Figure 2). He successfully underwent TIPS with the resolution of hematemesis. He was extubated on post-procedure day three and was transferred to the medical floor the following day.

**Figure 3:**
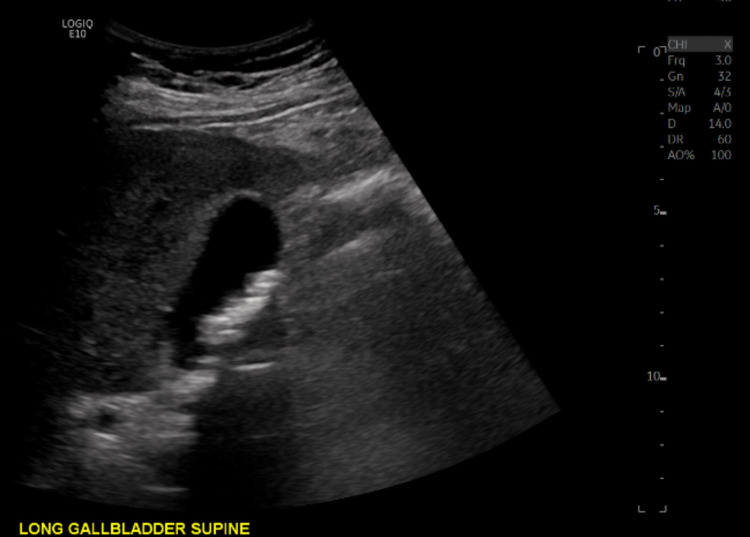
Liver ultrasound showing multiple, small mobile gallstones with no suspicious gallbladder wall thickening or pericholecystic fluid.

**Figure 4:**
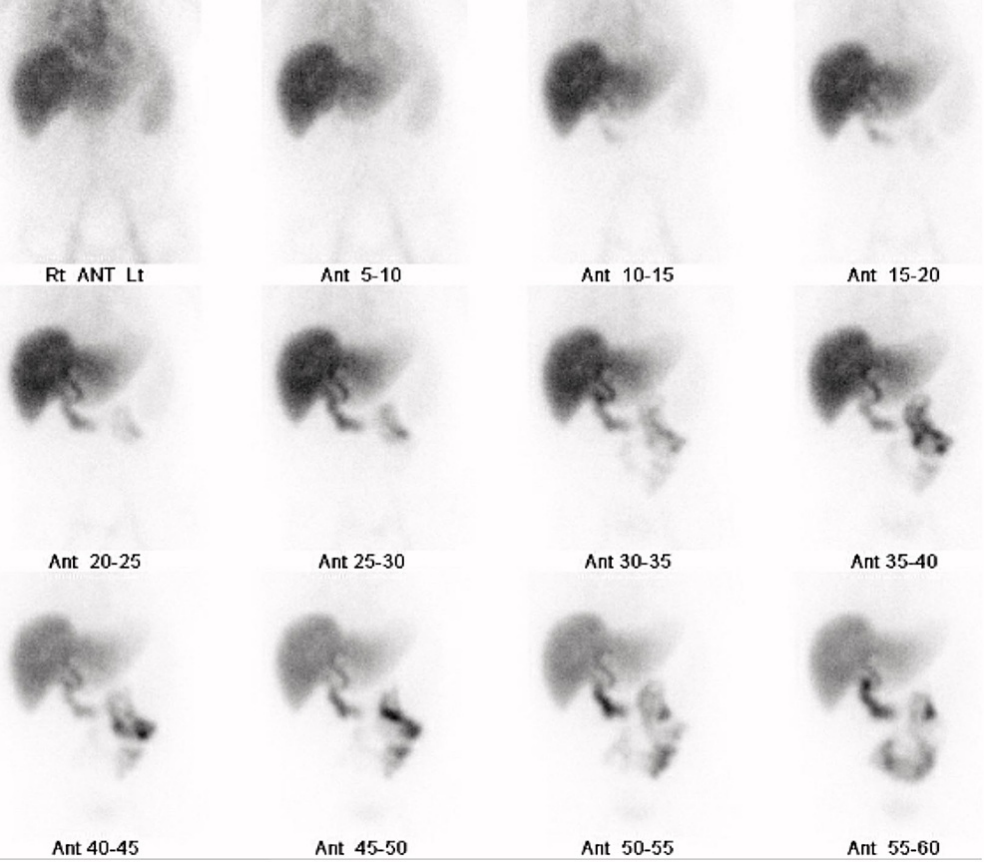
HIDA scan showing no evidence of cystic or common bile duct obstruction. Tracer is seen in the small bowel at 15 minutes. After morphine administration, tracer is seen in the gallbladder. HIDA - Hepatobiliary Iminodiacetic Acid

**Figure 5:**
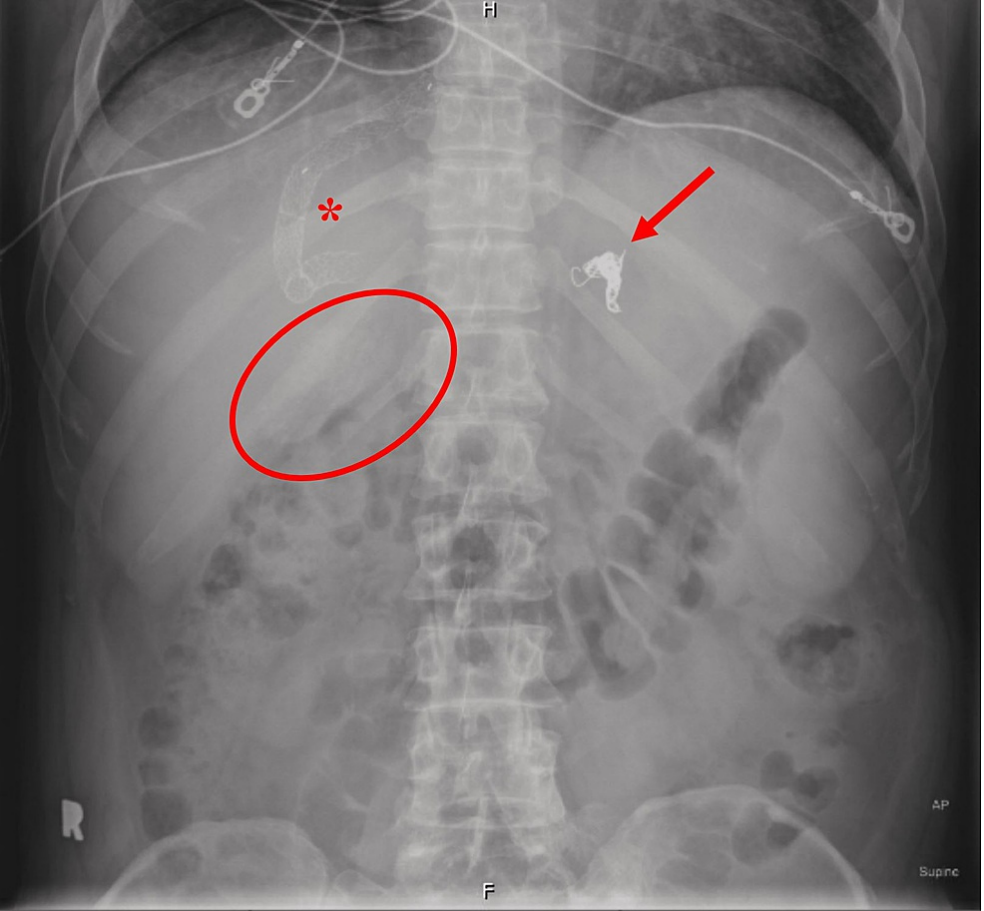
Abdominal radiograph showing diffusely radiopaque gallbladder (circle), a stent (asterisk) in the right upper quadrant, and embolization coils (arrow) in the left abdomen.

General surgery was consulted for management recommendations of incidental imaging findings of PG and EA. On bedside evaluation, the patient denied any fever, chills, dyspnea, abdominal pain, or changes in bowel function before the single episode of black, tarry stool. He had no history of prior colonoscopy. Besides the hematemesis, the patient felt at baseline without any additional complaints. Aspartate aminotransferase, alanine aminotransferase, alkaline phosphatase, and total bilirubin were elevated at 172 IU/L, 218 IU/L, 164 IU/L, and 3.7 mg/dL, respectively. Liver ultrasound was obtained during the patient’s prior hospitalization five months ago to evaluate the extent of alcoholic cirrhosis. The ultrasound showed multiple, small mobile gallstones with no suspicious gallbladder wall thickening, pericholecystic fluid, or common bile duct dilation (Figure 3). The patient’s transaminitis and elevated total bilirubin were likely secondary to his history of alcoholic cirrhosis and recent TIPS procedure; nevertheless, a hepatobiliary iminodiacetic acid (HIDA) scan was obtained, which showed no evidence of common duct or cystic duct obstruction (Figure 4). An acute abdomen series (chest and abdomen radiograph) was obtained the day before discharge to evaluate the pulmonary edema that was noted on the post-intubation radiograph, in conjunction with the recently placed TIPS stent. The abdominal radiograph showed diffuse gallbladder calcifications consistent with PG (Figure 5). Given the lack of abdominal pain and absence of acute cholecystitis, the patient was scheduled for follow-up with general surgery to evaluate his progress post-hospitalization. He was discharged two days after the general surgery consultation and continued to deny abdominal discomfort.

## Discussion

PG and EA are rare imaging findings in asymptomatic patients. A dilemma exists regarding whether incidental findings should be evaluated without other clinical indicators of disease. Prior retrospective reviews have shown that the rate of incidental CT imaging findings varies considerably, with only a fraction being clinically significant. For example, Sich et al. found incidental imaging findings in 70% of trauma patients (674 total cases reviewed), with only 36% of those being clinically relevant [7]. Veerappan et al., on the other hand, found incidental imaging findings in 46% of asymptomatic patients (2,277 total cases reviewed), with only 11% of those being clinically relevant [8]. Investigating incidental imaging findings can be expensive and Kimberly et al. estimated the average cost in 2008 at $248 per patient ($307 in 2021) [9]. Fear of missing a life-threatening diagnosis, fear of ligation, and medical facility protocols are all reasons for working up incidental findings, and the clinical benefit to the patient may be minimal [10].

EA is a rare imaging finding with an incidence of 8.8 per 1 million people [1]. Epiploic appendages are 0.5-5 cm long serosa-covered fat pads that abut the colonic wall and number 50-100 [1]. The exact role of epiploic appendages is unknown, with some reports suggesting they aid in immune function or colonic absorption [11]. Epiploic appendages are mainly located at the rectosigmoid junction (57%), followed by the ileocecal region (26%), ascending colon (9%), transverse colon (6%), and descending colon (2%) [12]. The pathophysiology of EA involves either torsion of an epiploic appendage or spontaneous venous thrombosis of a draining appendageal vein. Although the notion of inflammation of the epiploic appendages has been well-documented throughout the nineteenth century, the term ‘epiploic appendagitis’ was not coined until Dockerty et al. in 1956 [13]. Prior to the development of CT and magnetic resonance imaging in the 1970s, identifying EA as an acute cause of abdominal pain was difficult, and patients may have been subjected to unnecessary surgery without any significant intraoperative findings explaining the etiology of their pain, such as appendicitis, diverticulitis, and other causes of an acute abdomen [14]. CT imaging has become a fundamental tool to evaluate patients presenting with abdominal pain, and the imaging characteristics of EA are unique. Singh et al. described the CT appearance of EA as a fat-density ovoid structure adjacent to the colon that is usually 1.5-3.5 cm in diameter [15]. An alternative CT appearance of EA was presented by Almeida et al. as an ovoid object with a thin high-density rim, 1-3 mm thick, known as the hyperattenuating ring sign [16]. Identification of EA in a patient with abdominal pain can prevent unnecessary surgical intervention, as the disease is self-limited with symptom resolution in less than two weeks. The use of non-steroidal anti-inflammatory drugs (NSAID) can also be given to shorten the duration of symptoms [1].

PG is a rare imaging finding with an incidence of 0.06-0.08% of routine cholecystectomy specimens. The concept of ‘ceramic gallbladder’ was first described in 1959 by Cornell and Clark, but documentation of gallbladder wall calcification dates back to 1797 [17]. Early studies showed that the historical rate of gallbladder carcinoma in PG samples ranged from 7-61.5% [17]. As such, cholecystectomy was the treatment of choice in patients with PG to minimize the risk of gallbladder carcinoma. More recent literature suggests that the true incidence of gallbladder carcinoma in patients with PG is approximately 2-3% [18]. Two distinct types of gallbladder calcification have been reported: selective mucosal calcification and diffuse intramural calcification. Khan et al. reviewed pathology of 13 PG patients among 1200 cholecystectomy specimens [18]. Of those with PG, complete transmural calcification was found in 69% of specimens and mucosal calcification in 23% without any pathologic evidence of gallbladder carcinoma. In another study by Stephen and Berger, gallbladder carcinoma was only found in PG specimens with selective mucosal calcification [19]. This was postulated to be related to the destruction of the mucosal layer, resulting in devascularization and chronic inflammation. Similar to our patient, Khan et al. reported on three patients with incidental imaging findings of PG and one of those patients did not demonstrate PG on pathology [18]. Interestingly, Appel et al. completed a retrospective review of 133 patients with CT imaging findings of PG and described a CT false-positive rate of 32% when the imaging was read by different radiologists [20]. The rate of gallbladder carcinoma in this population was 6%, which was either confirmed using CT imaging or surgical pathology. None of the remaining patients with CT imaging confirmed PG developed gallbladder carcinoma at 6.6 ± 4.6 years follow-up.

## Conclusions

We described the case of a 54-year-old male with incidental imaging findings of EA and PG. No prior studies could be identified discussing imaging findings of both EA and PG in a single patient. Acute, focal abdominal pain is the most common presentation of EA and clinical correlation with CT imaging is essential to prevent unnecessary surgical intervention. The abdominal pain typically resolves spontaneously in less than two weeks without intervention and NSAID therapy can be used to shorten the duration of symptoms. The clinical presentation of PG can vary, however, patients usually exhibit signs consistent with chronic cholecystitis. Our patient denied any history of right upper quadrant abdominal pain that is classic of cholecystitis. The paradigm of treating patients with PG has shifted over recent years. Historically, all patients would undergo cholecystectomy to minimize the risk of gallbladder carcinoma. The association between PG and gallbladder carcinoma may be lower than previously reported and conservative management with serial gallbladder ultrasounds is acceptable, especially in patients who are poor surgical candidates. This report discusses two important imaging findings in a single patient that may have traditionally steered a clinician towards surgery, but recent evidence now suggests that conservative management is appropriate.
